# Old Wives' Physic

**Published:** 1900-03-31

**Authors:** John Beddoe


### Hospital Clinics and Medical Progress.

### OLD WIVES' PHYSIC.

By John Beddoe, M.D., F.R.S., F.R.C.P.

The origin of primitive, or, as some may prefer to call it, old wives' physic, is a subject of considerable psychological interest, and even of some practical importance.

Accordingly, we find that many of those popular remedies which we now, rightly or wrongly, look upon with ridicule, were, two or three generations ago, employed by learned and thoughtful physicians. Thus when we laugh at the nurse who tells us her patient cannot be in danger from inanition, because he takes plenty of good clear soup, or of beeftea that the spoon will stand up in when it is cold, we should recollect that the physicians of a hundred years ago (and indeed some not so very far back) entertained the same erroneous belief, and that eminent French chemists thought so highly of the dietetic value of gelatin that they held a pound of bone equal in nutritiveness to six pounds of meat.

Again, some popular therapeutic practices which have never obtained a footing among our faculty may nevertheless be founded on accurate observation and sound reasoning, The story of the discovery of the virtues of chinchona bark by a fever-stricken Indian of the Orinoco may likely enough be true. The Australian blacks, much as we despise their intellectual capacity, have found out for themselves an excellent surgical remedy for snakebite, when it is located on the leg, as it usually is. They tie a ligature tightly round the limb above the wound, and then scarify the limb pretty deeply in a circle between the wound and the ligature. One would almost think they had some idea of the circulation of the blood. Thirdly, a very large number of such practices were based on superstitions, religious or astronomical. And some were founded on some fancied resemblance, relation, or analogy between the disease and the supposed remedy. Thus, certain substances of a yellow colour, e.g., rhubarb, were imagined to be useful in jaundice.

The authors of the old herbals were, in most cases, men of some reading and classical learning; they knew what Galen and Dioscorides had written of the plants they described. Thus old Gerard never fails to tell us whether any particular herb or root is hot or cold, moist or dry, and the greater part of his specific directions are drawn from classical physicians. But there were in later times other manuals of domestic medicine which were more of an empiric character. Such a one was John Wesley's "Primitive Physick." It professed to afford "an easy and natural method of curing most diseases," and must have been almost as popular as his hymns and other theological works, for it attained to its thirty-sixth edition in 1849, which is probably more than was ever reached by any work of any legitimate physician. Wesley had not, apparently, studied Galen or Dioscorides, as Gerard had, but he had read Boerhaave, Sydenham and Huxham, and occasionally quoted them and a few other physicians; but their prescriptions formed but a small part of his repertory; and it is only in "complications" or manifestly dangerous or severe cases that he recommends his reader to apply to "a physician who fears God." Wesley's rules for diet and regimen are excellent, as might be expected from a delicate and laborious man who himself attained an advanced age. Some of them were "transcribed from Dr. Cheyne," the famous Bath physician. Thus, "Water is the wholesomest of drinks, quickens the appetite and strengthens the digestion most." "We may strengthen any weak part of the body by constant exercise: thus, the lungs by loud speaking, or by walking up an easy ascent" (this is doubtless true of the heart), and "digestion and the nerves by riding."

Wesley was a most zealous advocate of cold bathing, which, of course, was not in his day commonly practised, as it is among us. Not only did he recommend it to be used by weakly persons in order to acquire or retain good health; he also prescribed it for the cure of many diseases. He even related the case of a "Mrs. Bates of Leicestershire," obviously the kind of person who nowadays would make the fortune of an advertising quack. This lady "was cured of cancer in the breast, a consumption, a sciatica, and rheumatism, which she had near twenty years," by cold bathing daily for a month! She reminds one of the perversely-surnamed Mrs. Maria Jolly, who was cured of "fifty years' indescribable agony" by a box of Holloway's pills.

Wesley was far ahead of Hahnemann as regards both the pathology and the treatment of scabies. In fact, he was in advance of his time in this respect. "This distemper," he says, "is nothing but a kind of very small lice, which burrow under the skin. Therefore inward medicines are absolutely needless. Is it possible any physician should be ignorant of this?" Of the applications he recommends, three at least are really good, viz.: 1, sulphur; 2, black soap; 3, powder of white hellebore, mixed with cream, and used as an ointment. Others, such as ointment of powdered ginger, and a strong decoction of hyssop, may possibly be of some use, but are never tried. Stavesacre is not mentioned. Yet Gerard knew from Dioscorides that it was useful for destroying lice. But, in truth, Gerard was not apparently aware that scabies was a distinct disease, and accordingly he mentions various plants, such as elecampane, potamogeton, stavesacre,and palma Christi, as being serviceable outward applications; while the orobus (bitter vetch), the heart's-ease, senna, tamarinds, were to be taken internally for the cure of the "itch, leprosy, and tetters."

Common treacle was in favour with Wesley, both as an internal medicament and as an external application. It is one of his remedies for a common cold; and it may be noted that the Hindus are of his opinion: a coolie, who has been exposed to coldand wet, eats a quantity of gur (coarse black sugar), in order to avert the consequences, Treacle posset was a favourite domestic remedy fifty years ago.

It is difficult to realise that so late as the middle of the last century, alkalies, as such, were almost unknown in medicine. In popular or old wives' practice decoctions of various plants, containing alkaline salts, seem to have taken done their place,and their office. Miss Williams has shown that in the case of culinary vegetables these salts mostly exude into the broth in cooking. Accordingly, the juice or decoction of nettles was in great favour. Wesley even thought that it would delay for years the progress of old age towards decrepitude. In my early days this plant was in great popular repute as an anti-scorbutic.

One would perhaps expect a religious reformer to be ready to accept novel ideas or remedies in medicine, or, in plain English, to be new-fangled. And Wesley was so. The science of electricity was in its earliest infancy; but he gives a list of forty-five diseases or morbid conditions which he believed to be curable by "electrifying." The list, however, of those curable by cold bathing was even longer; and a third most potent remedy, truly an old wife's one, was "fasting spittle."

